# The influence of race tactics for performance in the heats of an international sprint cross-country skiing competition

**DOI:** 10.1371/journal.pone.0278552

**Published:** 2022-12-09

**Authors:** Pål Haugnes, Jan Kocbach, Rune Kjøsen Talsnes, Dionne Noordhof, Gertjan Ettema, Øyvind Sandbakk

**Affiliations:** 1 Centre for Elite Sports Research, Department of Neuromedicine and Movement Science, Faculty of Medicine and Health Sciences, Norwegian University of Science and Technology, Trondheim, Norway; 2 NORCE Norwegian Research Centre AS, Bergen, Norway; 3 Department of Sports Science and Physical Education, Nord University, Bodø, Norway; Universita degli Studi di Verona, ITALY

## Abstract

The purpose of this study was to examine the influence of race tactics for performance in the heats of an international sprint cross-country (XC) skiing competition in the classical style. Thirty elite male XC skiers (age: 24±3 years, sprint International Ski Federation [FIS] points: 61±27) performed a sprint time-trial (STT) followed by one to three ‘knock-out’ heats on a 1.7 km racecourse. An integrated GNSS/IMU system was used to determine position, sub-technique distribution and kinematics. Positioning was analysed using the television broadcast of the race. STT rank correlated positively with the final rank [(*r*_*s*_ (28) = .72, *P* = .001)]. The top-two finishers in each heat were on average ~3.8% slower in the heats compared to the STT (237.1±3.9 vs. 228.3±4.0 seconds, *P* = .001). On average, the skiers performed ~10 overtakings per 100 meters from the start to the last uphill segment but only ~3 overtakings per 100 meters in the last two segments in each heat. 93.8% of the top-two finishing skiers positioned themselves at top 2 before approaching the final uphill, in which the top-two finishers and the skiers ranked 3–4 were generally faster than those ranked 5–6 in the heats (both, *P* = .01). Here, top-four skiers employed 5.3% longer cycle lengths and 3.4% higher cycle rates in the diagonal sub-technique than skiers ranked 5–6 (all, *P* = .01). The present study demonstrates the importance of race tactics for performance in the heats of sprint XC skiing, in which the main performance-determining factors in the present racecourse were a front position when approaching the final uphill segment combined with the ability to ski fast in that segment. In general, this illustrates how accurate racecourse analyses may help skiers to optimize their race-individual race-strategies in the heats of sprint XC skiing competitions.

## Introduction

An International Ski and Snowboard Federation (FIS) regulated sprint cross-country (XC) skiing competition consists of one to four ~3-minute races performed on varying terrain over a 1.0- to 1.8-km racecourse separated by ~15–60 minutes periods of recovery. The skiers employ different sub-techniques (gears) of the classical and skating styles, in which they continuously change between gears and adapt according to the terrain, speed, and race conditions [1, 2]. Sprint competitions begin with an individual qualifying sprint time-trial (STT), from which the thirty fastest skiers qualify for the subsequent ‘knock-out’ heats (i.e., five quarterfinals [QF], two semi-finals [SF], and one final [F]). In the heats, six skiers compete head-to-head using a knockout system where the top-two finishers in the QFs and SFs qualify for the subsequent heat along with the two fastest remaining skiers (‘lucky losers’). Consequently, skiers in the heats need to pace and position themselves to reach a top-two finish and then recover rapidly to perform well in the subsequent heat. Hence, sprint XC skiing requires well-developed aerobic and anaerobic energy delivery capacities, strength, and speed abilities, as well as technical and tactical expertise [3–11].

In the individual STT, the skiers’ pacing strategy (i.e., the individual’s distribution of metabolic energy) is regulated according to their own racing template while aiming to optimise the overall time based on the given conditions [2, 12–20]. However, the main aim in the subsequent heats is not necessarily to produce the fastest time, but to finish ahead of other competitors and thereafter recover rapidly for the subsequent heat. In heats, the skiers’ speed, positioning, and sub-technique selection depends on several factors additional to metabolic energy regulation, such as the other competitors’ race strategies, the advantage of drafting behind competitors, and avoidance of accidents. These factors may influence the skiers’ grade of fatigue, sprint abilities and position when reaching the finish sprint [21–27].

Thus far, only three recent studies have examined performance during the heats in sprint XC skiing [28–30]. Andersson et al. [29] found on average ~2.4% faster times in the heats than in the STT of a simulated skating sprint race, with most heat winners (61%) being positioned first already after the initial 30 meters of their respective heats. However, this simulated race monitored only a limited number of participants (twenty out of thirty male and fourteen out of thirty female skiers) with significant heterogeneity in performance level. Therefore, the fastest skiers were positioned first already from the start and could control the heats, unlike international sprint races where different types of race tactics are normally employed [11, 17]. In addition, Andersson et al. [29] classified sub-techniques and the corresponding kinematic patterns by using video recordings only in the finish sprint of the racecourse and in short uphill sections spanning 25–40 m, in which the few cycles analysed reduces the validity of their results. In two studies of Marsland et al. [28, 30], individual differences in pacing strategy, sub-technique selection, and related cycle characteristics were identified for female skiers over the entire racecourse of a classical sprint XC skiing race. However, there were too few competitors for the QFs to be held and only six out of twelve skiers were analysed. Consequently, the influence of pacing strategy and race tactics on performance in the heats of an actual sprint XC skiing race is not fully explored and therefore limits our current understanding of sprint XC skiing. This could be achieved by combining video analysis of positioning with the use of wearable sensor technology, allowing full-course analyses of speed and continuous detection of sub-technique selection and related kinematic patterns [1, 2, 19, 28, 30–44]. Therefore, the present study was designed to examine the influence of race tactics for performance in the heats of an international sprint cross-country (XC) skiing competition in the classical style.

## Materials and methods

### Participants

Thirty male XC skiers (age: 24±3 years, sprint FIS points: 61±27) that qualified for the knock-out heats an international FIS-regulated classical sprint XC skiing competition were selected for this study. The skiers’ performance level ranged from Tier 3 (National Level) to Tier 5 (World-Class Level) as defined by McKay et al. [45]. The competition analysed was held at the start of the competitive season (mid-November) in Norway in 2017. The study was pre-approved by the Norwegian Centre for Research Data (NSD), and all skiers were fully informed about the nature of the study before they provided their written consent to participate.

### Design

All skiers performed the individual qualifying STT followed by the knockout heats on a 1.7 km racecourse. First, we investigated how performance in the STT correlated with the final rank of the race. To determine the association between speed profiles, positioning, sub-technique selection and kinematics with performance in the heats, all skiers were monitored by an integrated GNSS/IMU system while the skiers’ positioning in the heat was analysed using the television broadcast of the race.

In addition, we did two complementary analyses: 1) we examined the difference in speed profiles and sub-technique selection between the heats and the STT in seven pre-selected skiers with high performance level (age: 27±4 years, sprint FIS points: 33±25) and thereby large chances to qualify for the subsequent heats (see S1 Appendix and Fig 5), and 2) as an indication of the generalizability of our results, we compared the number of overtakings performed in the analysed competition (performed in 2017) with a comparable FIS-regulated classical sprint XC skiing competition in 2019 by male skiers of a similar performance level (age: 25±3 years, sprint FIS points: 57±40) (see S2 Appendix).

### Instruments and materials

Position, speed, and movement data of all skiers were continuously measured using an integrated sensor device consisting of a global navigation satellite system (GNSS) and an inertial measurement unit (IMU) (Optimeye S5, Catapult Sports, Melbourne, Australia), using a sampling frequency of 10-Hz for GNSS data and 100-Hz for IMU data. GNSS lock was ensured by placing the sensor device in a clear outdoor space for a minimum of 10 minutes prior to the data collection to allow for the acquisition of satellite signals, and the sensor device was carried in a pocket in the upper part of the race bib. The sensor device has been validated for position, speed, and time analysis in XC skiing against a geodetic, multi-frequency receiver by Gløersen et al. [46], ensuring that the sensor device is able to reliably detect differences in performance in line with the research questions in the current study. The racecourse was measured using a high-end differential, multi-frequency and multi-GNSS receiver (Alpha-G3T, Javad, San Jose, CA, USA) to provide a valid racecourse and elevation profile. The skiers’ data were adapted to the standard racecourse for analysis of sub-technique selection and kinematic pattern. The sensor device failed to register GPS-data and caused missing data in one case (N = 1). One skier broke his pole twice in the same heat, and another skier fell, which caused missing data in two other cases (N = 2).

**Fig 1:**
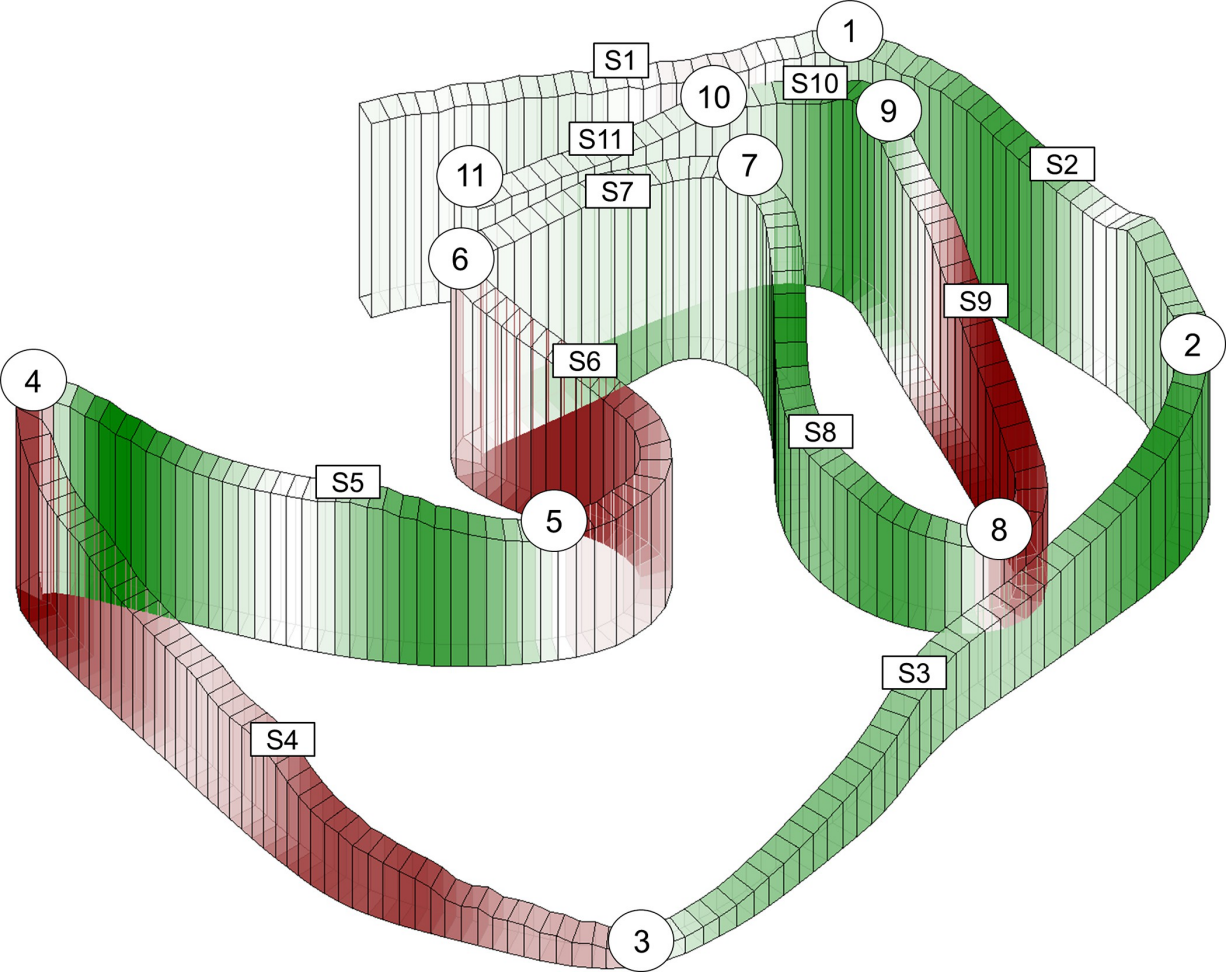
Racecourse. Three-dimensional illustration of the 1.7-km racecourse examined in the current study where the 11 segments (S1-S11) and 11 checkpoints are shown along the racecourse from start to finish. The colours represent terrain; uphill (red), flat (white), downhill (green), respectively.

Video analysis of positioning and accidents (obstruction, fall, pole break) were performed by using publicly available television broadcast of the Norwegian Broadcasting Corporation (NRK). For this analysis, the racecourse was separated into 11 segments (S1-S11) using 11 checkpoints according to terrain topography (Fig 1). Accordingly, these segments were grouped in the analysis of comparing racing time in the STT with the heats, where the various phases of the race were divided into the following groups: S1-S3, S4-S5, S6-S7, S8-S9, and S10-S11. The racecourse was 1720 m (consisting of 1 lap) and uphill, flat, and downhill made up 31%, 17%, and 52% of the total racecourse, respectively. The racecourse consisted of 3 uphill segments (S4, S6, and S9) with mean inclines of ~8%, 9%, and 9% and length of 280, 90, and 160 m; 3 flat segments (S1, S7, and S11) with length of 80, 110 and 100 m; 5 downhill segments (S2, S3, S5, S8, and S10) with mean slopes of approximately -4%, -5%, -3%, -7%, and -4% and length of 160, 300, 190, 170 and 80 m. The maximal elevation difference was 21 m with a total climb of 44 m for the entire racecourse. The last uphill in the racecourse in segment S9 consisted of a technique zone, where only the diagonal stride (DIA) was allowed to use. Obstruction was classified according to the following classification criteria: Skier A suffered loss of speed and/or rank due to skier B performing track change irregularly. Prior to the race, the skiers performed self-selected warm-up procedures according to their own individual program. All skiers used ski equipment optimised for the specific athlete’s racing preferences, including poles, boots, and skis. All ski-base preparations, including grinds, structure, and waxing, were optimised for the snow conditions on the competition day by the team of each individual. The weather conditions were stable throughout the entire day, with light wind, partly cloudy, air and snow temperature of -3°C, ~84% humidity, and atmospheric pressure of ~901.6 hPa. The racecourse was covered by hard-packed artificial snow, which had been machine-prepared the morning of the race day.

### Sub-technique classifications

The sub-technique classification was undertaken using a K-Nearest Neighbour algorithm as previously described by Solli et al. [44]. The algorithm was trained on nine skiers for the racecourse used in this competition and seven skiers for another racecourse, and subsequently tested on three other skiers with a per-distance classification accuracy of 96% for the complete racecourse in this competition. The classifications were also examined visually by comparing a graphical representation of filtered accelerometer and gyroscope signals with those typical for the various sub-techniques. Any errors in the automated classification were subsequently corrected based on the visual inspection. The error for GPS-measured time vs. the official time in the race was 0.05±0.18 seconds. The sub-techniques were classified as diagonal (DIA), double poling with a kick (DK), double poling (DP) and Other, the latter including tuck (TCK) and various turning techniques (TRN). The cycles were automatically segmented based on peak detection of Gaussian low pass filtered data from one axis of the gyroscope. Cycle length (CL) was calculated as the average speed multiplied by the cycle time (CT) and the cycle rate (CR) was calculated as the reciprocal of cycle time.

### Statistical analysis

All data are presented as mean ± standard deviation (SD), unless otherwise stated. Shapiro-Wilk test, visual inspection of Q-Q plots, and comparison of histograms were used to assess normality. Levene’s test was used to assess the homogeneity of variances. The Spearman rank-order correlation coefficient was performed to assess the relationship between the STT rank and the final rank. A paired-samples t-test was used to determine whether there was a statistically significant difference between the race time in STT and the heats for the top-two performers in each heat, and race time in STT vs. the heats in different segments for seven skiers analysed in detail (see S1 Appendix). Positioning for heat winners and top-two finishers were explored. During the heats, the percentage of heat winners’ and top-two finishers’ checkpoint positioning (i.e., rank 1–6) was determined. Positioning of all skiers was examined by assessing the relationship between intermediate rankings and final rankings by using Kendall tau-b correlations. To assess the repeatability of overtakings between 2017 vs. 2019 we used the intraclass correlation coefficient (ICC), a two-way random-effect model based on single measurements and absolute agreement. Percentage difference equals the absolute value of the change in value, divided by the average of the 2 number, all multiplied by 100. A one-way ANOVA was conducted to determine if speed and kinematics for DIA in S9 were different for groups with different final ranking (heat rank 1–2, heat rank 3–4, heat rank 5–6). Tukey post-hoc test was used to assess the significance of differences between groups. The magnitude of the correlation coefficients and effect sizes were interpreted as following: 0.0–0.1, trivial; 0.1–0.3, small; 0.3–0.5 moderate; 0.5–07, large; 0.7–0.9, very large; 0.9–1.0, nearly perfect [47]. The statistical significance level was set at α*<*0.05. All statistical analyses were processed using STATA 16.0 software (Stata Corporation, College Station, TX, USA) and Office Excel 2016 (Microsoft Corporation, Redmond, WA, USA).

## Results

### The relationship between STT rank and the overall ranking

There was a large positive correlation between STT rank and the final rank of the race, [*r*_*s*_(28) = .72, *P* = .001].

### Speed, time, positioning, and technique distribution

Skiing times for the top-two finishers and number of incidents (obstruction, fall, pole break, yellow card) are shown in Table 1. Top-two finishers were on average ~3.8% slower in the heats than in the STT [average heat time: 237.1±3.9 vs. STT: 228.3±4.0 (s)], with the difference being significant [*t*(9) = -6.578, *P* = .001, *d* = 2.22]. The number of overtakings on the different segments included in total 124 overtakings during the heats (i.e., ~16 overtakings in each heat). By adjusting overtakings for segment length, it becomes clear that the skiers performed ~10 overtakings per 100 meters from the start to the final uphill segment (S2 to S9), and only ~3 per 100 m on the two last segments (S10 to S11). Most overtakings were observed in the longest uphills (S4 and S9), although many overtakings were also performed in all types of segment types (i.e., uphill, downhill, and undulating terrain).

**Table 1:** Skiing time for the top-two finishers and number of incidents during a 1.7-km classical sprint cross-country skiing race for elite male skiers. Presented as absolute values [N = 16].

Variable	QF 1	QF 2	QF 3	QF 4	QF 5	SF 1	SF 2	F
**Heat rank 1 (s)**	236.00	235.00	238.10	237.90	243.90	233.20	232.40	230.40
**Heat rank 2 (s)**	236.50	235.40	238.70	238.20	244.10	233.40	232.40	231.50
**Heat rank 1 (%STT**_**top1**_ **)**	6.9	6.5	7.7	7.6	9.9	5.8	5.4	4.6
**Heat rank 2 (%STT**_**top1**_ **)**	7.1	6.6	7.9	7.7	10.0	5.8	5.4	5.1
**Obstruction**	3	2	1	3	0	3	1	1
**Fall**	0	0	0	1	0	0	0	0
**Pole break**	0	0	0	0	2	0	0	0
**Yellow card**	0	0	0	0	0	0	0	0

Abbreviations: STT, time-trial; QF, quarterfinal; SF, semi-final; F, Final; %STT_top1_, percentage of the sprint time-trial winner time.

**Fig 2:**
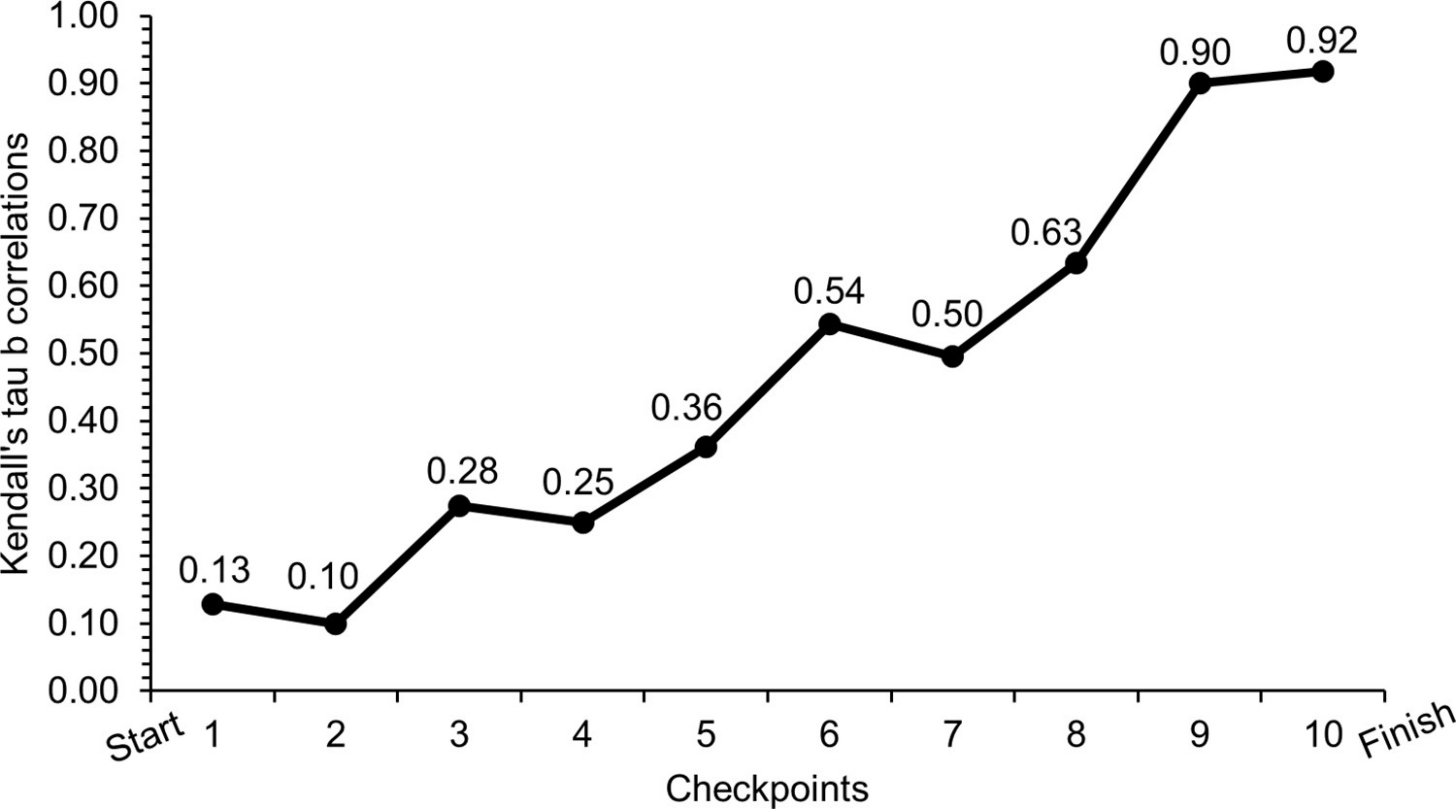
Kendall’s tau-b correlations. The relationship between intermediate ranks and final rank during the heats of a classical sprint cross-country skiing race for elite male skiers [N = 30].

The positioning of heat winners and top-two finishers at different checkpoints during the racecourse are presented in Table 2. Here, 75% of those who won the heats were positioned first in the final uphill segment, whereas 93.8% of the top-two finishers were being positioned first or second on this segment. The relationship between intermediate ranks and final ranks during the heats is presented in Fig 2. As expected, the strength of the correlation increased segment-by-segment throughout the race and was strongest at the end of the race.

**Table 2:** Percentage of heat winners (left part) and top-two finishers (right part) positioned at rank 1–6 at the 11 checkpoints during the heats of a 1.7-km classical sprint cross-country skiing race for elite male skiers. Presented as percentages [N = 16].

Variable	Heat winner						Top 2-finishers					
Checkpoints 1–11	Rank 1	Rank 2	Rank 3	Rank 4	Rank 5	Rank 6	Rank 1	Rank 2	Rank 3	Rank 4	Rank 5	Rank 6
**1. Out of stadium**	25.0%	12.5%	25.0%	12.5%	12.5%	12.5%	12.5%	31.3%	18.8%	18.8%	6.3%	12.5%
**2. First downhill**	25.0%	12.5%	25.0%	12.5%	12.5%	12.5%	12.5%	31.3%	12.5%	18.8%	6.3%	18.8%
**3. End of first downhill**	50.0%	0.0%	12.5%	25.0%	12.5%	0.0%	31.3%	18.8%	12.5%	18.8%	12.5%	6.3%
**4. End of first uphill**	37.5%	12.5%	0.0%	25.0%	25.0%	0.0%	31.3%	25.0%	6.3%	12.5%	18.8%	6.3%
**5. End of second downhill**	37.5%	12.5%	37.5%	0.0%	12.5%	0.0%	31.3%	25.0%	25.0%	6.3%	6.3%	0.0%
**6. End of second uphill**	50.0%	12.5%	25.0%	0.0%	12.5%	0.0%	43.7%	25.0%	18.8%	6.3%	6.3%	0.0%
**7. Before third downhill**	75.0%	0.0%	12.5%	0.0%	0.0%	12.5%	50.0%	12.5%	31.3%	0.0%	0.0%	6.3%
**8. End of third downhill**	75.0%	0.0%	12.5%	0.0%	0.0%	12.5%	50.0%	25.0%	18.8%	0.0%	0.0%	6.3%
**9. End of third uphill**	75.0%	12.5%	12.5%	0.0%	0.0%	0.0%	43.8%	50.0%	6.3%	0.0%	0.0%	0.0%
**10. Before the finish-sprint**	75.0%	12.5%	12.5%	0.0%	0.0%	0.0%	43.8%	50.0%	6.3%	0.0%	0.0%	0.0%
**11. Finish**	100.0%	0.0%	0.0%	0.0%	0.0%	0.0%	50.0%	50.0%	0.0%	0.0%	0.0%	0.0%

Checkpoint 11 Finish: All heat winners were ranked 1, whereas top 2-finishers were either ranked 1 or 2.

**Fig 3:**
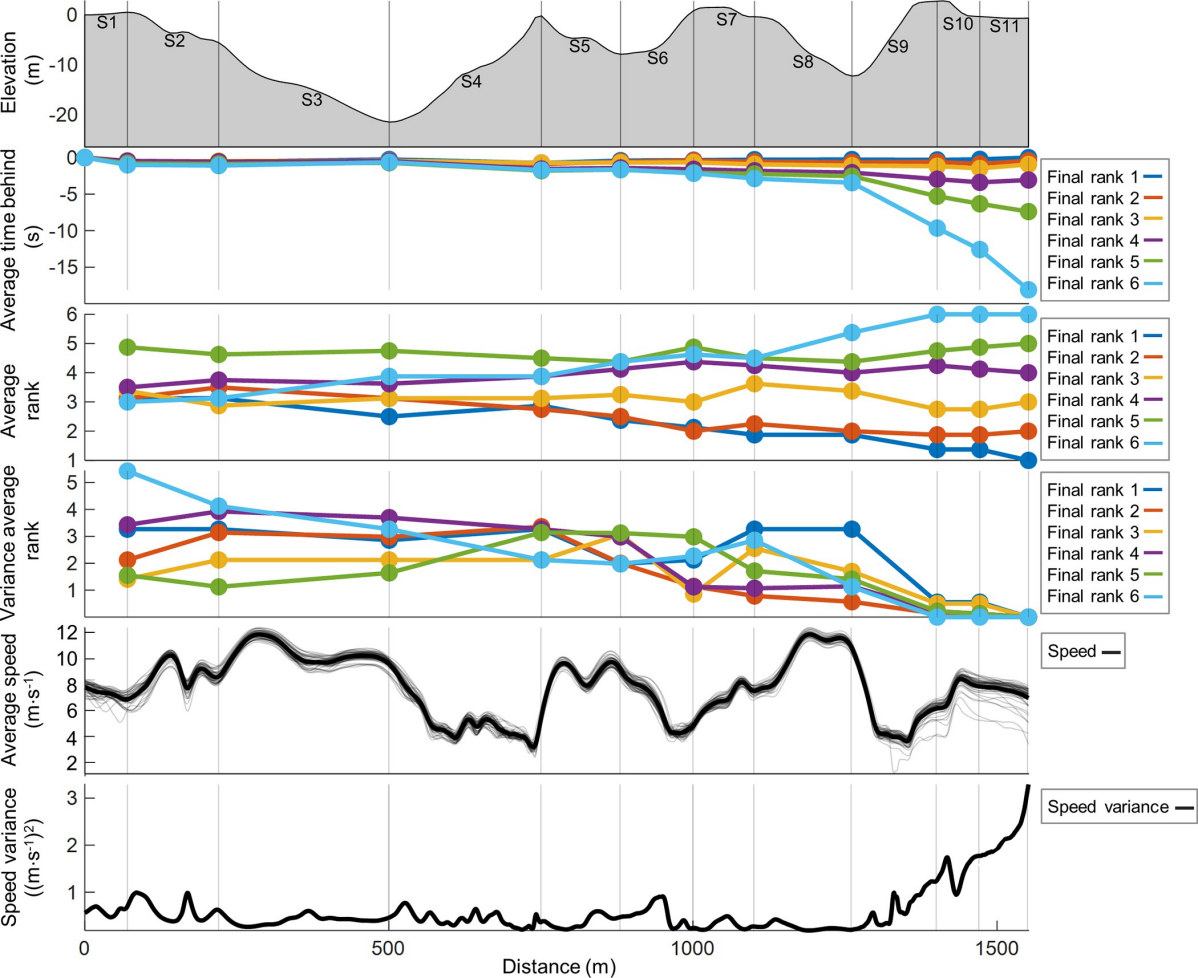
Time behind, speed, and positioning. The average time behind the winner, intermediate rankings, and skiing speed across the racecourse in 11 segments (S1-S11) during the heats of a classical sprint cross-country skiing race for elite male skiers [N = 29 time and speed, N = 30 positioning].

Fig 3 shows the average time behind the leader in each segment, as well as speed, and positioning for all heats. Here, all skiers were within 4 seconds behind the leader of the group until the final uphill in segment S9 where heat rank 1–4 were generally faster than heat rank 5–6 who were unable to keep up the pace and/or gave up.

**Fig 4:**
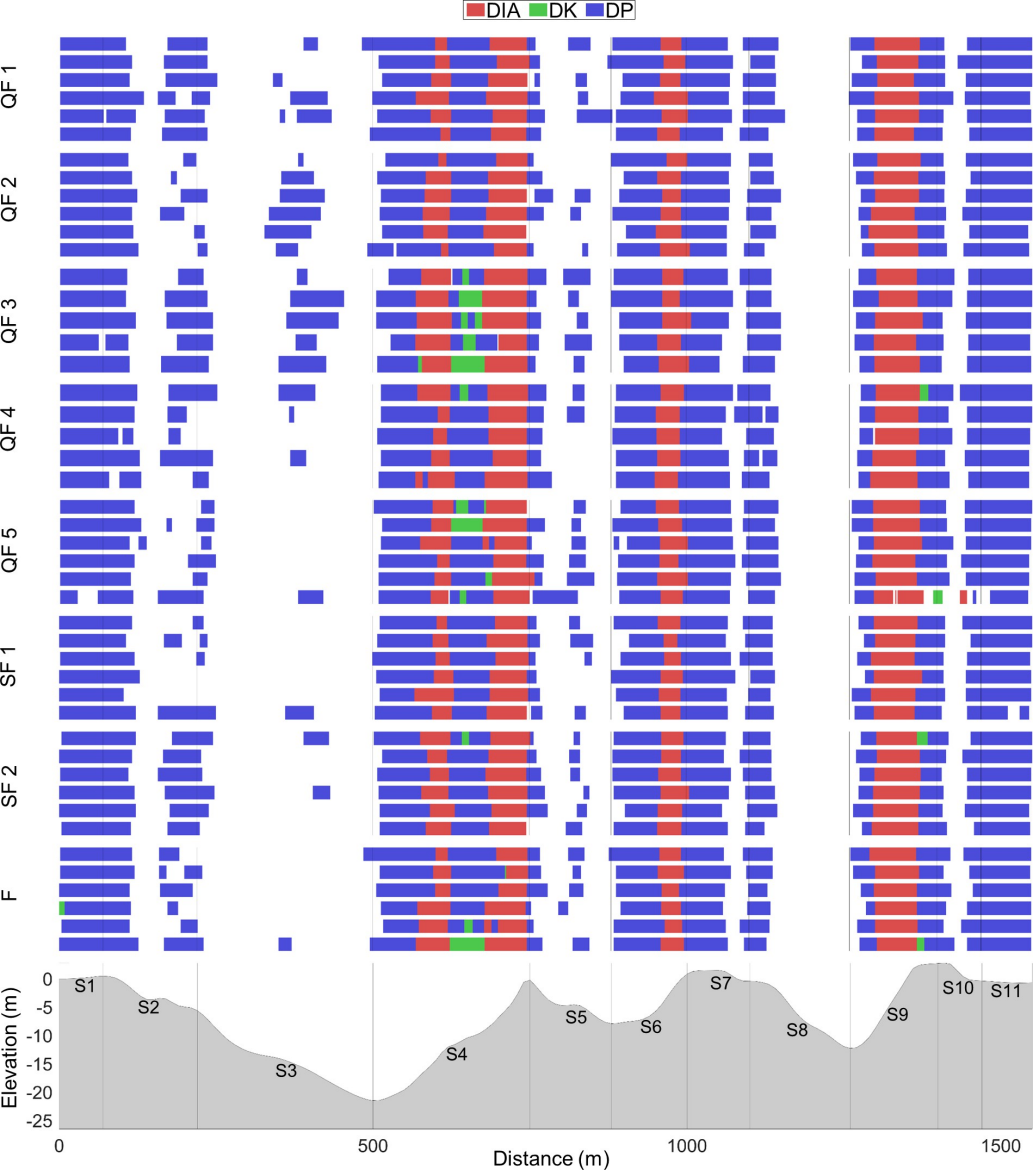
Individual sub-technique selection. The individual use of sub-techniques (diagonal stride [DIA], double poling with a kick [DK], double poling [DP]) and [Other] including tuck and turning techniques) across the racecourse in 11 segments (S1-S11) during the heats of a classical sprint cross-country skiing race for elite male skiers. The skiers are presented according to the order of ranking from top 1^st^ to bottom 6^th^ [N = 28].

**Fig 5:**
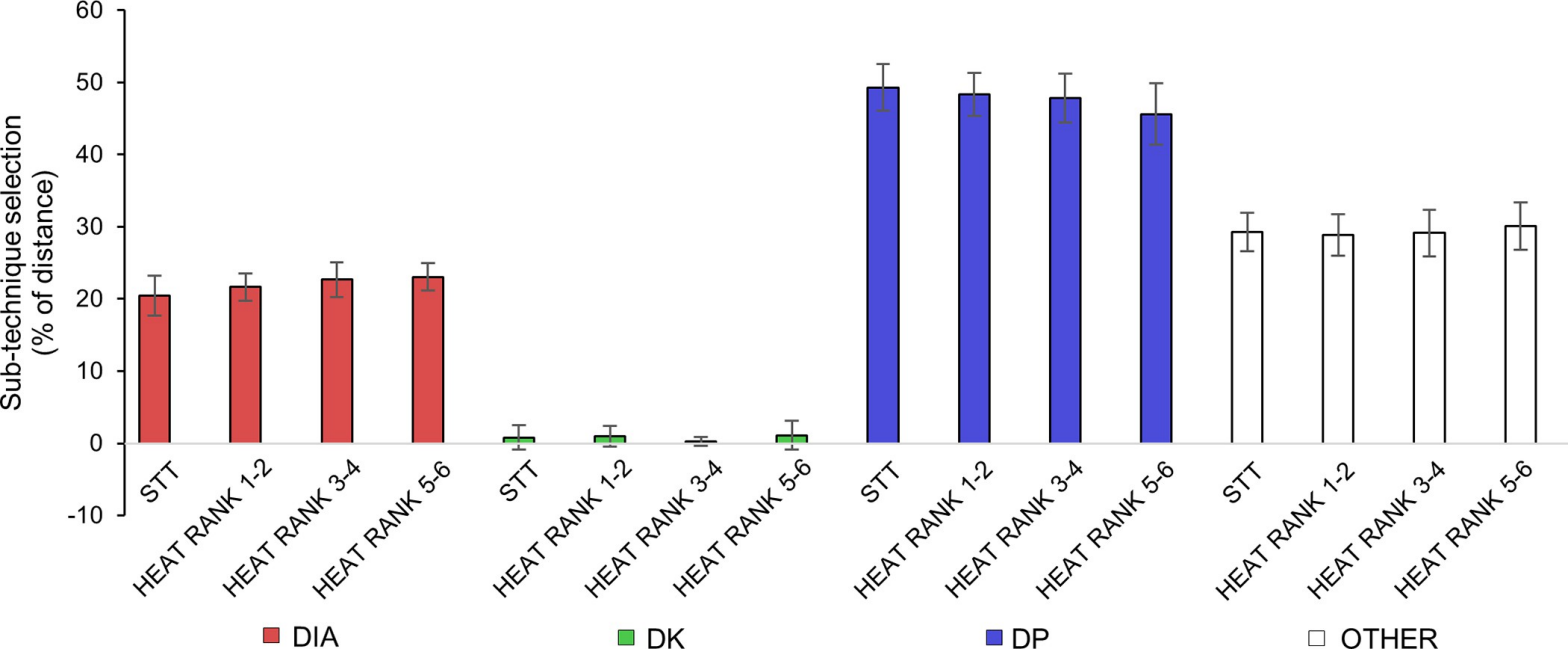
Sub-technique selection in relation to distance. The percentage use of sub-techniques (diagonal stride [DIA], double poling with a kick [DK], double poling [DP]) and ([Other] including tuck and turning techniques) in relation to distance across the racecourse during the sprint time-trial and heats of a classical sprint cross-country skiing race for elite male skiers. Error bars indicate SD [N = 28].

The distribution of sub-techniques in relation to the racecourse for all skiers during QFs, SFs and F are shown in Fig 4. Here, the skiers are presented according to the order of ranking in each heat, and all skiers demonstrated similar sub-technique selection along the racecourse. The percentage use of different sub-techniques in relation to the distance for the STT (N = 7) and heats (N = 28) is shown in Fig 5. Here, you can see that there is no statistical difference in the use of sub-technique selection.

Speed, CL, and CR for DIA in the last uphill segment (S9) are shown in Table 3. Here, skiers were grouped according to their overall rankings; heat rank 1–2 (N = 16), heat rank 3–4 (N = 16) and heat rank 5–6 (N = 13). The segment speed differed significantly between groups [*F*(2,42) = 32.32, *P* = .001, ω2 = 0.58], with the skiers ranked 1–2 and 3–4 showing significantly higher speed than those ranked 5–6 (both, *P* = .001), while the corresponding difference between heat rank 1–2 and 3–4 did not differ (*P* = .600). CL differed significantly between groups, [*F*(2,42) = 7.35, *P* = .002, ω2 = 0.22], with the skiers having heat rank 1–2 and 3–4 showing a significantly longer CL than those ranked 5–6 (*P* = .003 and .007). In contrast, the difference in CL between heat rank 1–2 and 3–4 did not differ (*P* = .936). CR differed significantly between groups, [*F*(2,42) = 7.02, *P* = .002, ω2 = 0.21], with the skiers having heat rank 1–2 and 3–4 showing a significantly higher CR than those ranked 5–6 (*P* = .003 and .012), while the difference between heat rank 1–2 and 3–4 was non-significant (*P* = .838).

**Table 3:** Cycle characteristics in the diagonal sub-technique during the final uphill segment in a 1.7-km classical sprint cross-country skiing race for elite male skiers. Values are mean ± SD [N = 27].

Variable	Heat rank 1–2	Heat rank 3–4	Heat rank 5–6
**Speed (m∙s** ^**-1**^ **)**	4.15 ± 0.12^c^	4.08 ± 0.18^c^	3.62 ± 0.25^a,b^
**CL (m)**	1.45 ± 0.08^c^	1.44 ± 0.08^c^	1.35 ± 0.07^a,b^
**CR (Hz)**	1.43 ± 0.05^c^	1.42 ± 0.06^c^	1.34 ± 0.10^a,b^

Abbreviations: CL, cycle length; CR, cycle rate. The letters indicate statistically differences from heat rank 1–2 (a), heat rank 3–4 (b), or heat rank 5–6 (c) (*P* < 0.05).

## Discussion

The purpose of this study was to examine the influence of race tactics for heat performance during an international sprint XC skiing race in the classical style. The main findings were as follows: 1) a large correlation between STT rank and the final rank of the race was found; 2) the top-two finishers in each heat were on average ~3.8% slower in the heats compared to the STT, a difference that, based on the seven skiers examined in detail (S1 Appendix), appeared during the initial segments of the heats; 3) on average, ~16 overtakings occurred in each heat, with a clear reduction in number of overtakings per 100 m in the two last segments; 4) 93.8% of the top-two finishing skiers positioned themselves on 1^st^ or 2^nd^ place when approaching the final uphill, in which the top-two finishers and the skiers ranked 3–4 were generally faster than the skiers ranked 5–6 in the heats, by employing 5.3% longer CL and 3.4% higher CR in the DIA sub-technique; and 5) we observed relatively similar sub-technique selection across skiers within and between heats.

While race tactics have previously been shown as an important performance determinant in other endurance sports such as cycling [48–50], middle-distance running [51–53], and short-track speed skating [54–56], this is the first study to examine race tactics in an actual sprint XC skiing race. We found a significantly large correlation between STT rank and the final rank of the race. This finding is in line with Spencer et al. [8], who reported moderate to very large correlations between STT rank and final rank in both classical and skating races for both men and women. Nevertheless, the correlations shows that a relatively large portion of the variance in final rank was not explained by STT performance, which demonstrates the importance of performance in the heats where race tactics and the ability to master an entire day comprising multiple heats are important performance-determinants.

In the study by Andersson et al. [29], skiers were found to be ~2.4% faster in the heats than in the STT, with two-thirds of the heat winners being positioned first already after the initial segment of the race. These results are in contrast with the results of the current study, where the skiers were on average ~3.8% slower in the heats than in the STT, with 50% of the heat winners being positioned first after one-third of the heat, but with substantial changes in positioning throughout the remaining part of the course. The reason for this difference might be explained by the high degree of heterogeneity in performance level (sprint FIS points for men and women: 87±42 and 90±53) in the previous study by Andersson et al. [29], while our study included a more homogenous group of skiers. As a result, one can assume that the heat winners in Andersson et al. [29] were motivated to finish as top two in the relegation system and were able to control the heat from the leader position due to the large difference in performance level. In contrast, compared with the individually optimised pacing strategy in the STT, the skiers in our study chose to ski close to the leader of the group from start until the final uphill segment to position themselves strategically. This resulted in slower speed at the beginning of the heats compared to the STT, whereas this speed difference gradually levelled out and the skiers did not differ in speed from S6 to the end of the racecourse. The lower speed in the first part of the racecourse during heats likely enabled skiers to change position more frequently, with overtakings being performed in all types of terrain segments. However, most overtakings were observed in the two longest uphill segments (S4 and S9). Accordingly, positioning at the checkpoint at the start of the final uphill segment was a key determinant of heat performance, in which 93.8% of the top-two finishers positioned themselves on 1^st^ or 2^nd^ place, although one skier was able to win the race by skiing from last position in the beginning of the uphill. The highest ranked skiers (rankings 1–4) were generally faster in this uphill segment (S9) by utilizing longer CL and higher CR in the DIA sub-technique compared to the skiers ranked 5–6. However, even if the final uphill segment was crucial for heat performance in this study, different racecourses and snow conditions might affect the skier’s race tactics, and every race (and skier) will, to some degree, require different tactical decisions [57, 58], as exemplified for both sexes in the study by Andersson et al. [29].

Although better-performing skiers produced longer CL and higher CR in DIA in the last uphill segment, all skiers selected similar sub-techniques during the race as seen with both the individual use of sub-techniques and the percentage use of sub-techniques in relation to distance (Figs 4 and 5). These findings are, however, in contrast to those of Marsland et al. [30] who found larger variations in sub-technique selection during a classical sprint race. A plausible explanation for this might be the differences in topography and race strategy between the two studies. In addition, the fact that the skiers in the previous study by Marsland et al. [30] were women while our study sample included men only may to some degree explain this difference in sub-technique selection as shown previously in a classical distance race [44]. Furthermore, the skiers in the heats in sprint XC skiing are often grouped during the race, and the skiers in our study mainly used the same sub-technique as the skiers in front. Hence, the racecourse together with the sub-technique selection employed by the leader of the group will often influence the variation in technique distribution in the heats. Therefore, future research should investigate skiers’ technique distribution and corresponding kinematic patterns in different races and whether a larger variation in sub-technique usage can be expected in female compared to male skiers.

Whether the findings of this study are generalisable or would differ between sexes, environmental conditions, and racing styles are topics for further investigations. However, the high ICC (.77, *P* = .004) found between overtakings in the 2017 competition and the repeated competition two years later (see S1 Table in S2 Appendix), indicates that similar race tactics identified in this study are to be expected in other races performed on the same racecourse by skiers of a similar performance level if the environmental conditions are comparable.

Overall, the interpretation of the most-optimal strategies identified in this study is based on the best skiers self-selected tactics, which are adopted not only for the given racecourse and snow conditions but also according to their opponents and the individual skier’s strengths and weaknesses. Further exploration of speed profiles together with the number of overtakings and where these take place would contribute to better understanding of the underlying mechanisms of race tactics. Here, the utilization of sensor technology and the classification of intermediate rankings along the racecourse would increase the validity of the measurements. Therefore, we suggest that future studies should further explore the effectiveness and metabolic cost of typical elements used as race tactics during sprint XC skiing. Such insights would allow a deeper insight in the underlying mechanisms of race tactics in sprint XC skiing and could potentially help practitioners to optimise race strategies for different race formats.

## Conclusions

The findings in this study demonstrate that race tactics is an important determinant of heat performance in sprint XC skiing. Race times in the heats were longer than the corresponding times for the STT, which can be explained by lower speed in the beginning of each heat, thereby allowing for multiple overtakings in these segments. There was a clear reduction in the number of overtakings after the final uphill segment, where most top-two finishers were already positioned first or second. Accordingly, a top-ranked position (i.e., 1^st^ or 2^nd^ place) before the final uphill segment, in combination with higher uphill DIA speed by utilising longer CL and higher CR, were the main performance determinants in the heats. However, each racecourse is different, and the practical implications are that accurate racecourse analysis combined with an understanding of each skier’s strengths and weaknesses may be crucial factors for success in the heats of sprint XC skiing.

## Supporting information

S1 Appendix The difference in speed profile between the heats and the sprint time-trial in seven pre-selected skiers. (PDF)

S2 Appendix The number of overtakings during a repeated classical sprint cross-country skiing race for elite male skiers performed in the same racecourse in 2017 and 2019. (PDF)

S1 File Data and analyses conduced in this study. (XLSX)
