# Supplementary material for: The influence of race tactics for performance in the heats of an international sprint cross-country skiing competition
Source: PLoS One. 2022 Dec 9;17(12):e0278552. doi: 10.1371/journal.pone.0278552 (PMC9733856; doi:10.1371/journal.pone.0278552)
Supplement: S1 Appendix — (PDF) [file pone.0278552.s001.pdf]

The seven skiers examined in detail both in the STT and the heats were slower in the first parts of the heats compared to the STT; S1-3 [55.9±1.3 vs. 50.6±1.1 (s)] and S4-5 [70.8±1.1 vs. 66.2±1.7 (s)], with the difference being significant for S1-3 [t(6)= -7.738, P = .001, d = 4.54)] and significant for S4-5 [t(6)= -6.024, P = .001, d = 3.26)]. STT and heats segment times did not significantly differ during the middle part and the last parts of the racecourse; S6-7 [29.9±1.2 vs. 30.2±.9 (s), t(6) = -.934, P = .386, d = 0.27)], S8-9 [44.8±5.4 vs. 43.9±1.5 (s), t(6)= -0.398, P = .704, d = 0.24)] and S10-11 [23.0±1.9 vs. 21.4±0.6 (s), t(6)= -7.778, P = .466, d = .44)]. However, three of the examined skiers in the heats finished as number 5-6 in the QF.

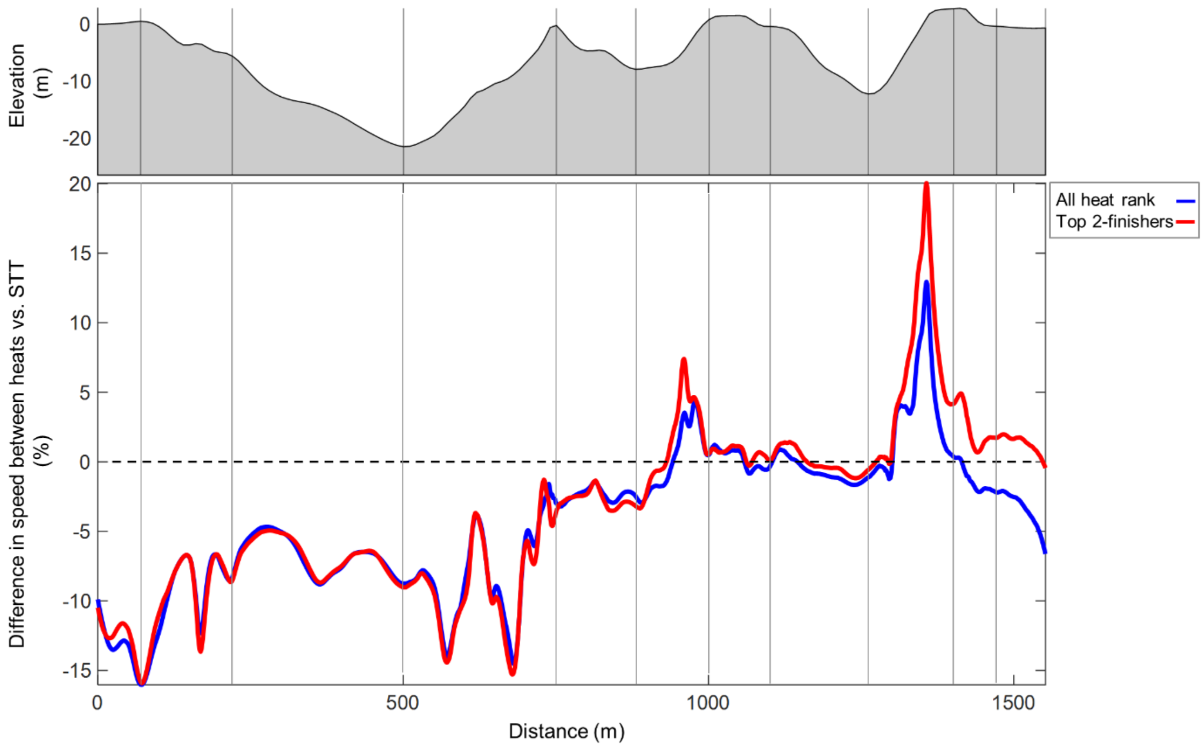

S1 Fig. Percentage difference. The difference in speed profile between the heats and the sprint time-trial in seven pre-selected skiers during a 1.7 km classical sprint cross-country skiing race for elite male skiers. [N=7 All heat rank, N=4 Top 2-finishers].
