# Supplementary material for: The influence of race tactics for performance in the heats of an international sprint cross-country skiing competition
Source: PLoS One. 2022 Dec 9;17(12):e0278552. doi: 10.1371/journal.pone.0278552 (PMC9733856; doi:10.1371/journal.pone.0278552)
Supplement: S2 Appendix — (PDF) [file pone.0278552.s002.pdf]

S1 Table. The number of overtakings during a repeated classical sprint cross-country skiing race for elite male skiers performed in the same racecourse in 2017 and 2019. Presented as absolute values [N=60].

Variable	QF 1		QF 2		QF 3		QF 4		QF 5		SF 1		SF 2		F		Total	
	2017	2019	2017	2019	2017	2019	2017	2019	2017	2019	2017	2019	2017	2019	2017	2019	2017	2019
**Checkpoint 1-2**	0	3	1	2	2	2	1	0	0	3	0	0	2	3	4	1	10	14
**Checkpoint 2-3**	5	4	2	1	3	5	1	4	1	5	2	0	4	1	1	0	19	20
**Checkpoint 3-4**	6	2	2	2	4	3	3	5	4	6	0	0	2	5	4	5	25	28
**Checkpoint 4-5**	1	1	2	0	3	0	1	0	2	0	3	0	1	0	0	0	13	1
**Checkpoint 5-6**	3	0	3	2	0	1	1	3	3	0	0	1	2	1	1	1	13	9
**Checkpoint 6-7**	0	1	0	1	3	0	5	1	2	1	1	0	0	3	0	2	11	9
**Checkpoint 7-8**	0	1	0	3	2	0	4	0	3	0	1	0	1	0	0	0	11	4
**Checkpoint 8-9**	0	2	1	3	2	4	2	5	7	0	2	2	1	5	2	4	17	25
**Checkpoint 9-10**	0	0	0	2	1	0	0	1	0	0	0	0	0	0	0	1	1	4
**Checkpoint 10-11**	0	0	0	1	0	0	0	0	2	1	0	1	1	0	1	1	4	4
**Checkpoint 1-11**	15	14	11	17	20	15	18	19	24	16	9	4	14	18	13	15	124	118

Abbreviations: QF, quarterfinal; SF, semi-final; F, Final
